# Evaluation of Three Antimicrobial Peptides Mixtures to Control the Phytopathogen Responsible for Fire Blight Disease

**DOI:** 10.3390/plants10122637

**Published:** 2021-11-30

**Authors:** Rafael J. Mendes, Sara Sario, João Pedro Luz, Natália Tassi, Cátia Teixeira, Paula Gomes, Fernando Tavares, Conceição Santos

**Affiliations:** 1 Faculty of Sciences, University of Porto, 4169-007 Porto, Portugal; sara.sario@fc.up.pt (S.S.); catia.teixeira@fc.up.pt (C.T.); pgomes@fc.up.pt (P.G.); ftavares@fc.up.pt (F.T.); csantos@fc.up.pt (C.S.); 2 LAQV-REQUIMTE, Biology Department, Faculty of Sciences, University of Porto, 4169-007 Porto, Portugal; 3 CITAB—Centre for the Research and Technology of Agro-Environmental and Biological Sciences, University of Trás-os-Montes e Alto Douro, 5000-801 Vila Real, Portugal; 4 CIBIO—Research Centre in Biodiversity and Genetic Resources, InBIO, Associated Laboratory, Campus Agrário de Vairão, University of Porto, 4485-661 Vairão, Portugal; 5 QRural, Polytechnic Institute of Castelo Branco, School of Agriculture, 6000-909 Castelo Branco, Portugal; j.p.luz@ipcb.pt; 6 LAQV-REQUIMTE, Department of Chemistry and Biochemistry, Faculty of Sciences, University of Porto, 4169-007 Porto, Portugal; natalia.tcpf@fc.up.pt

**Keywords:** alamarBlue reduction, AMPs, BP100, CA-M, *Erwinia amylovora*, flow cytometry, green revolution, hypersensitive response, RW-BP100, sustainable control

## Abstract

Fire blight is a severe bacterial plant disease that affects important chain-of-value fruit trees such as pear and apple trees. This disease is caused by *Erwinia amylovora,* a quarantine phytopathogenic bacterium, which, although highly distributed worldwide, still lacks efficient control measures. The green revolution paradigm demands sustainable agriculture practices, for which antimicrobial peptides (AMPs) have recently caught much attention. The goal of this work was to disclose the bioactivity of three peptides mixtures (BP100:RW-BP100, BP100:CA-M, and RW-BP100:CA-M), against three strains of *E. amylovora* representing distinct genotypes and virulence (LMG 2024, Ea 630 and Ea 680). The three AMPs’ mixtures were assayed at eight different equimolar concentrations ranging from 0.25 to 6 μM (1:1). Results showed MIC and MBC values between 2.5 and 4 μM for every AMP mixture and strain. Regarding cell viability, flow cytometry and alamarBlue reduction, showed high reduction (>25%) of viable cells after 30 min of AMP exposure, depending on the peptide mixture and strain assayed. Hypersensitive response in tobacco plants showed that the most efficient AMPs mixtures and concentrations caused low to no reaction of the plant. Altogether, the AMPs mixtures studied are better treatment solutions to control fire blight disease than the same AMPs applied individually.

## 1. Introduction

*Erwinia amylovora* is a highly destructive quarantine plant pathogen of pome fruit trees [e.g., apple (*Malus domestica*) and pear (*Pyrus communis*)], and other wild and ornamental plants, causing the fire blight disease [1,2,3]. Although widely distributed worldwide (e.g., USA, Canada, UK, Portugal, France, Italy, South Korea, New Zealand, and Middle East), there is still a lack of proper control measures of this plant pathogen [4,5,6].

Current control measures are mainly based on preventive action against fire blight (cultural control, such as, pruning of early-stage infected trees for example), and/or copper-based phytosanitary compounds and antibiotic applications (chemical control), which is prohibited in the European Union with a few exceptions, due to the dramatic rise of antibiotic resistance [7,8,9,10,11]. Alternative sustainable control methods of *E. amylovora* have been explored extensively in the last few years, such as, the use of antagonistic bacteria [12], bacteriophages [13], essential oils [14], and antimicrobial peptides (AMPs) [15,16,17].

AMPs, also known as host defense peptides, are defined as natural polypeptide sequences, composed of cationic and hydrophobic amino acids (2 to 50) with antimicrobial activity [18]. They are part of the innate immune system of diverse organisms, such as, animals and plants [19]. The data repository of antimicrobial peptides (DRAMP) has recently reported to contain 22,259 entries, and 5909 candidate AMPs to control several microorganisms [20]. AMPs have been gaining interest as a promising sustainable tool to control phytopathogenic bacteria due to their low toxicity to the host plant, and predominantly for the fact that they have low propensity to induce bacteria-acquired resistance [21,22,23].

Structurally, AMPs are categorized in five different subgroups, depending on their amino acid sequences, net charge and protein structure: anionic AMPs, cationic α-helical AMPs, β-sheet AMPs, extended cationic AMPs, and fragments from antimicrobial proteins [19]. Based on their mode of action, AMPs are classified in two models: the transmembrane pore model, which includes both barrel-stave pore and toroidal pore; and nonpore models (e.g., carpet model) [18,19]. AMPs act by binding to bacterial membranes, which is initially driven by the electrostatic attraction between the AMPs’ cationic residues and the negatively charged components of the lipidic outer membrane; some peptides have the capacity to penetrate bacterial cells and further interact with nucleic acids and other intracellular targets [18].

Previous studies have emphasized the bacteriostatic and/or bactericidal action of several AMPs against different plant pathogenic bacteria, namely, *Xylella fastidiosa* [24], *Pseudomonas syringae* pv. *actinidiae* [25,26], *Pseudomonas syringae* pv. *syringae*, *Xanthomonas arboricola* pv. *pruni*, *Xanthomonas fragariae*, *Xanthomonas axonopodis* pv. *vesicatoria* [25], and *E. amylovora* [17,25,27].

The effects of AMPs on *E. amylovora* and their use as a control measure for this bacterium have been reported in several studies over the last 15 years [16,27,28,29,30,31]. Recently, we have demonstrated the bioactivity of two AMPs against *E. amylovora* [17], namely, RW-BP100 and CA-M, which had already been reported to display activity against pathogenic bacteria but never tested against the causative agent of the fire blight disease [32,33,34]. RW-BP100 is an analogue of BP100, a well-known AMP presenting high antimicrobial activity against *E. amylovora*, and low phytotoxicity and haemolysis [27,28,35], and CA-M is a cecropin A-melittin (CA-M) hybrid peptide, also known as CA(1–7)M(2–9).

Although most studies on the potential application of AMPs against phytopathogenic microorganisms have been focused on the use of individual peptides [36,37,38,39], several works have been recently assessing mixtures of AMPs with other bactericidal components, such as, lysozyme [40], antibiotics [41], and other different AMPs [15,26,42]. Even though the latter has been showing promising results, up to date there is only one study that has explored the mixture of different AMPs, namely, peptides flg15 and BP16, to control the phytopathogen *E. amylovora* [15].

In connection with the above, the aim of this work was to study the putative synergic effect of three AMPs mixtures (BP100, RW-BP100, and CA-M) which were previously shown to be particularly promising against *E. amylovora* when tested individually [17]. To this end, three different AMPs mixtures at different equimolar concentrations were used against three different strains of the pathogen representing distinct genotypes and virulence (LMG 2024 as the type strain, Ea 630 a highly virulent strain, and Ea 680 a mild virulent strain [6]. Several susceptibility indicators and viability assays were analyzed, complemented with *in planta* bioassays to disclose the effect of these AMPs mixtures on *E. amylovora*.

## 2. Results

### 2.1. Peptide Synthesis

The three peptides, namely, BP100, RW-BP100 and CA-M (Table 1) were synthesized by standard solid-phase peptide synthesis protocols based on the Fmoc/tBu orthogonal protection scheme [43]. The peptides were isolated with high purity (>98%), according to reverse-phase high performance liquid chromatography (RP-HPLC) analysis, and their expected molecular weights (MW) were confirmed by electrospray ionization-ion trap mass spectrometry (ESI-IT MS) (Figures S1–S6).

### 2.2. AMPs Antimicrobial Activity

The MIC, MBC and IC_50_ values of the AMPs mixtures against two *E. amylovora* strains (Ea 630 and Ea 680) and the type strain LMG 2024 were determined (Table 2). The MIC values ranged between 2.5 and 4 μM for both B:C and R:C mixtures, whilst 2.5 μM was obtained for B:R against the three tested strains. The MBC values ranged between 3.25 and 4 μM for B:C and R:C mixtures, while for B:R they were equal to the MIC values (2.5 μM) whatever the strain.

**Figure 1:**
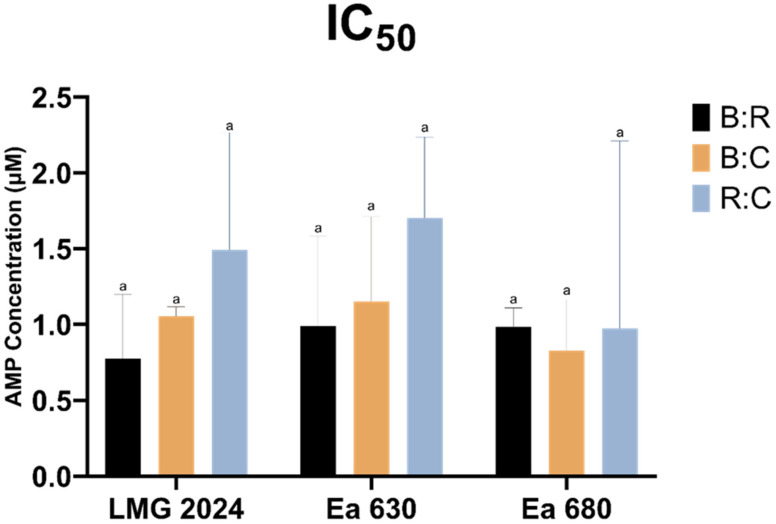
Half maximal inhibitory concentration (IC_50_) of three *Erwinia amylovora* strains against three AMPs mixtures: BP100:RW-BP100 (B:R), BP100:CA-M (B:C), and RW-BP100:CA-M (R:C). Vertical bars: mean value with standard deviation (n = 3). Different letters denote significant differences (*p* < 0.05).

Since some AMPs mixtures presented the same MIC and MBC values, IC_50_ was determined in order to further investigate the susceptibility of the strains to the tested peptides (Figure 1). Although no statistically significant differences (*p* > 0.05) were observed between the strains in each AMPs mixture, a slightly different susceptibility for each strain was detected regarding the tested mixtures, allowing us to distinguish the strains regarding their IC_50_. The lowest IC_50_ value against strains LMG 2024 and Ea 630 were obtained with the B:R mixture (0.77 ± 0.42 and 0.98 ± 0.59 μM, respectively), whereas the mixture B:C presented the lowest IC_50_ (0.82 ± 0.33 μM) against strain Ea 680. Furthermore, it was possible to observe that the R:C mixture presented generally higher IC_50_ values and the B:R mixture lower ones.

### 2.3. Viability Determination after Exposure to AMPs Mixture

#### 2.3.1. Flow Cytometry Assessment of Viability

**Figure 2:**
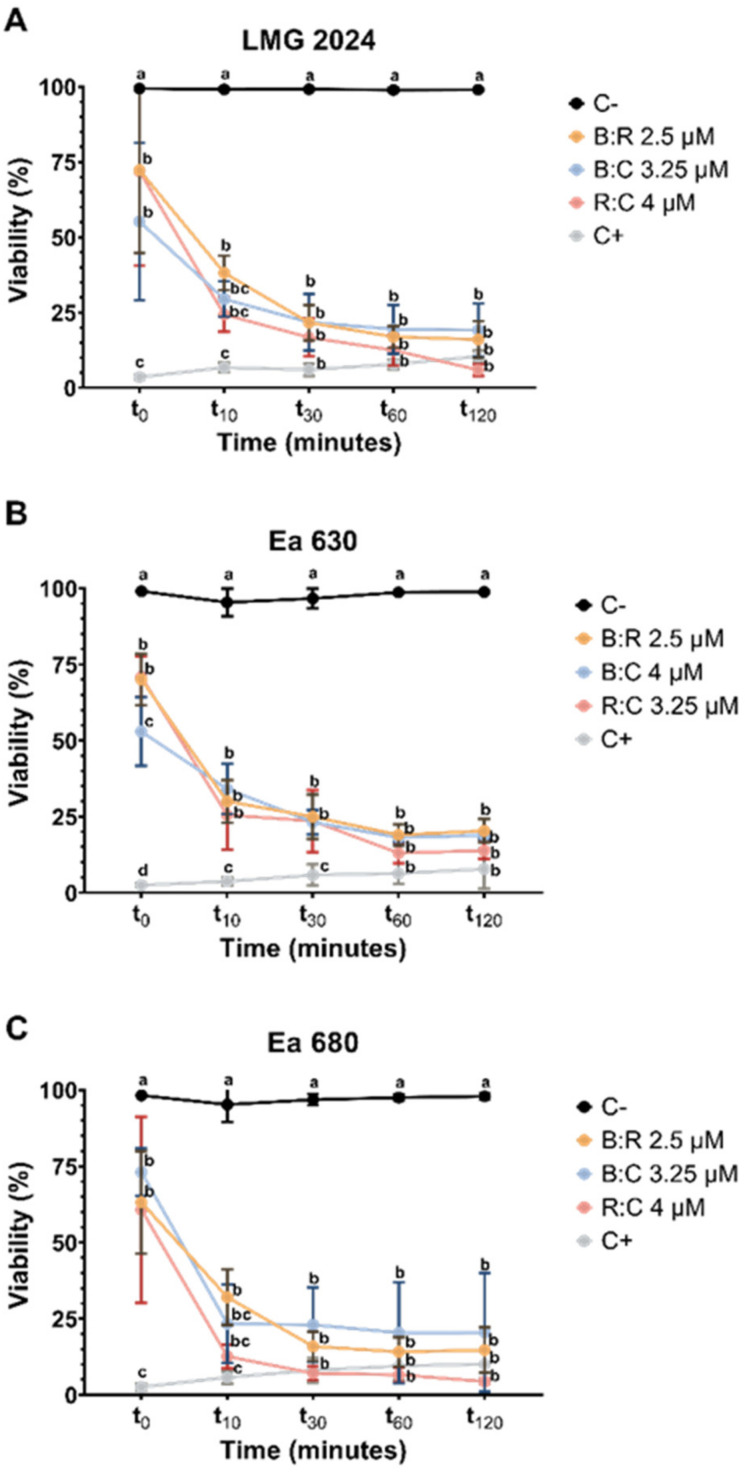
Viability evaluation through flow cytometry of three *Erwinia amylovora* strains after exposure to different concentrations of AMPs mixtures. (**A**) LMG 2024; (**B**) Ea 630 and (**C**) Ea 680. C−: negative control; C+: positive control. Vertical bars: mean value with standard deviation (n = 3; different letters denote significant differences (*p* < 0.05).

To evaluate *E. amylovora* viability after exposure to different AMPs mixtures, FC was applied in order to quantify the loss of membrane integrity caused by the peptides. The percentage of viability was inversely proportional to PI fluorescence (Figure 2).

The three combinations of AMPs, tested at concentrations corresponding to their respective MBC values, caused a statistically significant reduction comparing with negative control (*p* < 0.05) of cell viability (55% to 72%) on type strain LMG 2024 after initial exposure (t_0_) with a decrease below 50% being achieved after 10 min of exposure to the AMPs mixtures (24% to 38%) (Figure 2A). Furthermore, it was possible to observe that after 1 h of exposure to the peptides, type strain LMG 2024 viability decreased (17% to 12.5%), and stabilized up to 2 h of exposure, except for R:C at 4 μM, which reduced furthermore the viability (6%). Regarding strains Ea 630 (Figure 2B) and Ea 680 (Figure 2C), the results followed a similar trend as for strain LMG 2024, i.e., after initial exposure, cell viability decreased significantly comparing with negative control (*p* < 0.05) (59% to 71% and 60.7% to 73%, respectively), decreasing below 50% after 10 min of exposure to all AMPs mixtures, especially strain Ea 680 (32% to 12.6%). After 2 h of exposure, viability decreased between 13% to 18.9% (Ea 630) and 6.6% to 14% (Ea 680). Overall, all AMPs mixtures led to a statistically significant reduction in cell viability comparing with negative control (*p* < 0.05) for the three strains throughout all time points. Comparing the three tested strains, Ea 680 showed the lower viability values at every time point from t_10_ to t_120_.

**Figure 3:**
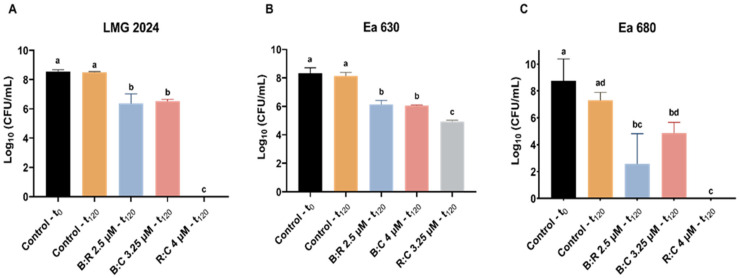
Number of viable cells of three *Erwinia amylovora* strains after treatment with different concentrations of AMPs mixtures for FC assay. (**A**) LMG 2024; (**B**) Ea 630; (**C**) Ea 680. Vertical bars: mean value with standard deviation (n = 3). Different letters denote significant differences (*p* < 0.05).

CFU plating count was performed for all strains after 2 h of exposure to AMPs mixtures, to further assess cell viability after treatment (Figure 3). All strains displayed a statistically significant decrease (*p* < 0.05) in CFU (between 0 and 4.77 × 10^6^ CFU·mL^−1^, 8.59 × 10^4^ and 1.58 × 10^6^ CFU·mL^−1^, and 0 and 1.85 × 10^5^ CFU·mL^−1^, for strain LMG 2024, Ea 630 and Ea 680, respectively) when comparing with the control (between 2.61 × 10^8^ and 3.46 × 10^9^ CFU·mL^−1^). Noteworthy was that, after 2 h exposure of strains LMG 2024 and Ea 680 the R:C mixture, no growth of the bacteria was observed (Figure 3A,C). After treatments with B:R and B:C, the total number of colonies obtained for strain LMG 2024 decreased significantly to 4.7 × 10^6^ CFU·mL^−1^ and 3.5 × 10^6^ CFU·mL^−1^, respectively (Figure 3A). Moreover, all tested mixtures led to a significant reduction in CFU for strain Ea 630 (1.6 × 10^6^, 1.1 × 10^6^, and 8.6 × 10^4^ CFU·mL^−1^, for B:R, B:C and R:C, respectively) when compared to the control (Figure 3B). For strain Ea 680, when treated with B:R and B:C, a decrease in CFU (5.1 × 10^3^ and 1.9 × 10^5^ CFU·mL^−1^, respectively) was also observed (Figure 3C).

#### 2.3.2. AB Reduction Viability Assessment

**Figure 4:**
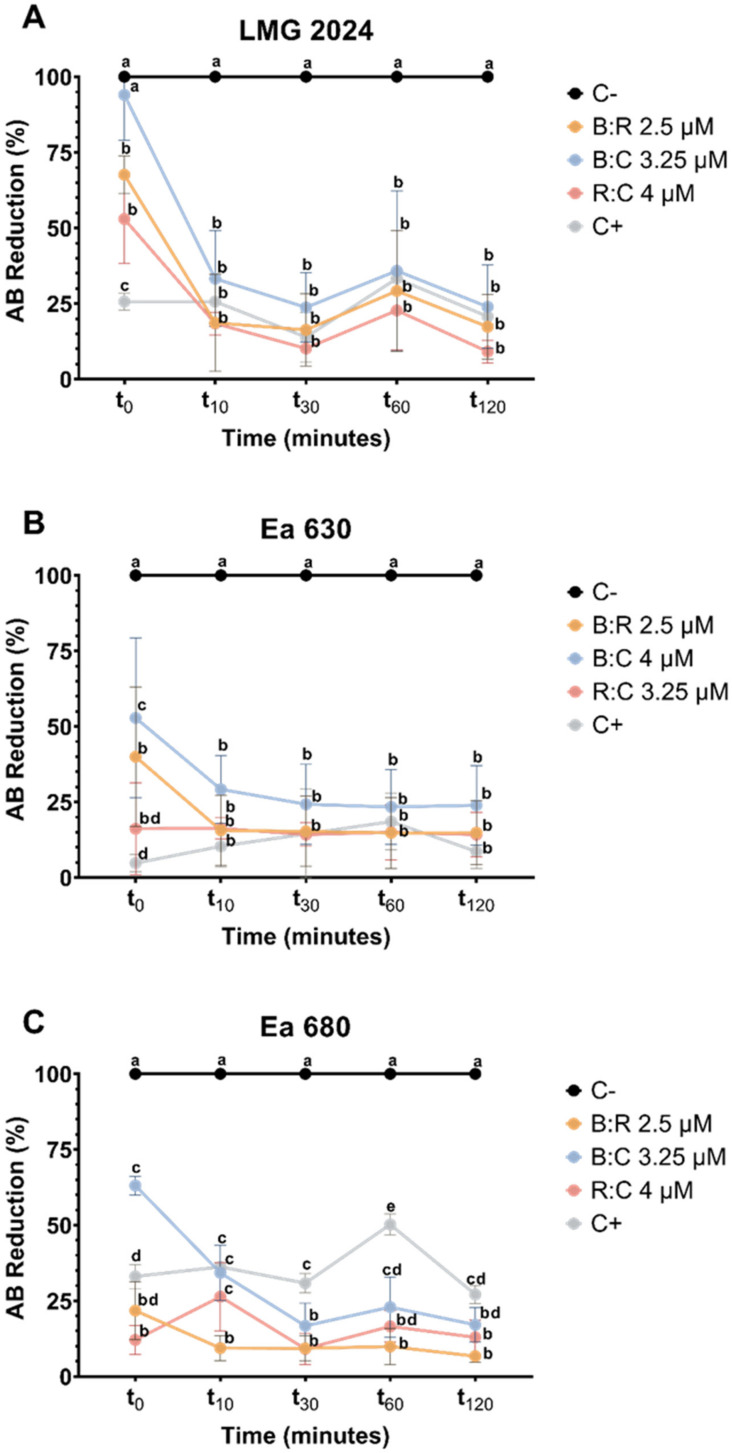
Viability evaluation through alamarBlue™ of three *Erwinia amylovora* strains after exposure to different concentrations of AMPs mixtures. (**A**) LMG 2024; (**B**) Ea 630 and (**C**) Ea 680. C−: negative control; C+: positive control. Vertical bars: mean value with standard deviation (n = 3). Different letters denote significant differences (*p* < 0.05).

In order to get further insight into cell viability after exposure to the three AMPs mixtures, the percentage of AB reduction was determined (Figure 4). Viability of cells is directly proportional to the reduction of AB, which is caused when metabolic active cells convert resazurin to resorufin.

Results show that at the time of exposure (t_0_), the AMPs mixtures led to a statistically significant decrease comparing with negative control (*p* < 0.05) in viability for the three strains tested, except for LMG 2024 when tested against B:C (Figure 4A). After 10 min of exposure to each peptide mixture, type strain LMG 2024 viability decreased below 50%, and continued to decrease until 30 min of exposure (values decreased between 10.7% to 23.7%). After 2 h of exposure, it decreased to values ranging from 9.1% to 23%. Regarding strains Ea 630 and Ea 680, both had cell viability reduced to values below 50% with each AMPs mixture after 10 min of exposure. Cell viability of strains Ea 630 and Ea 680 decreased throughout all time points of exposure to each AMPs mixture, with values between 23.9% and 14.2%, and 17.1% and 6.6%, respectively (Figure 4B,C).

**Figure 5:**
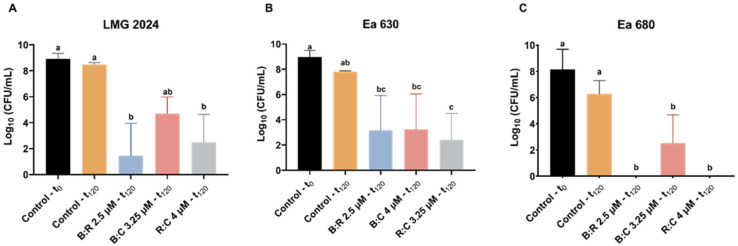
Number of viable cells of three *Erwinia amylovora* strains after treatment with different concentrations of AMPs mixtures for alamarBlue™ assay. (**A**) LMG 2024; (**B**) Ea 630 and (**C**) Ea 680. Vertical bars: mean value with standard deviation (n = 3). Different letters denote significant differences (*p* < 0.05).

CFU plating count method was applied to further investigate the cell viability after the AB reduction assay (Figure 5). Comparing with the control, the number of cells of every strain reduced statistically (*p* < 0.05) when exposed to the AMPs mixture, with LMG 2024 having fewer colonies when exposed to B:R (7.22 × 10^3^ CFU·mL^−1^) (Figure 5A). Strain Ea 630 had a higher reduction when compared to the control when exposed to R:C (2.83 × 10^3^ CFU·mL^−1^) (Figure 5B). For strain Ea 680, exposure to B:C led to a reduction of CFU to 3.86 × 10^3^ CFU·mL^−1^, while no growth of the bacterium was observed when exposed to B:R and R:C (Figure 5C).

### 2.4. Hypersensitive Response to AMPs

**Figure 6:**
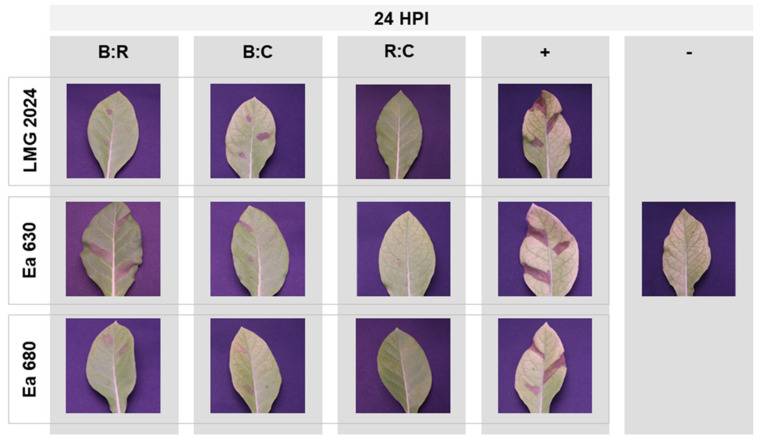
Hypersensitive response in tobacco leaves after 24 h of inoculation of three *Erwinia amylovora* strains exposed to three AMPs mixtures: BP100:RW-BP100 (B:R); BP100:CA-M (B:C); RW-BP100:CA-M (R:C); +: positive control; −: negative control (PBS); HPI: hours post infection.

Tobacco leaf infiltration assays were employed to assess the HR of the three strains of *E. amylovora* tested after exposure to the three AMPs mixture (Figure 6). Upon infiltration with LMG 2024 type strain, it was possible to observe that the exposure to B:R and B:C produced low and medium HR, respectively (severity scale of 1 and 2) when compared with the positive control (Figure 6, top line). Strain Ea 630, when exposed to B:R, produced a similar HR response to its positive control (severity scale of 3), whilst when exposed to B:C showed medium to weak HR response (severity scale of 1–2) (Figure 6, middle line). Lastly, infiltration with strain Ea 680 produced medium HR (severity scale 2) when treated with B:R and B:C AMPs (Figure 6, bottom line). When treated with the mixture R:C, none of the three strains were able to induce a HR response on tobacco leaves (severity scale 0) (Figure 6, third column), since no differences were observed to the negative control. HR assays were performed with each peptide individually against type strain (Figure S1).

**Figure 7:**
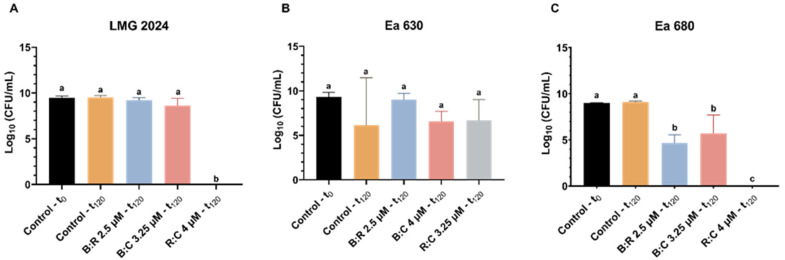
Number of viable cells of three *Erwinia amylovora* strains after treatment with different concentrations of AMPs mixtures for hypersensitive response in tobacco assay. (**A**) LMG 2024, (**B**) Ea 630, (**C**) Ea 680. Vertical bars: mean value with standard deviation (n = 3). Different letters denote significant differences (*p* < 0.05).

When performing CFU plate counting after inoculation of the tobacco leaves for the HR assay, it was possible to observe that after 24 h of inoculation in KB medium there were no viable cells of strains LMG 2024 and Ea 680 after exposure to R:C (Figure 7A,C). Furthermore, the number of viable cells of strain Ea 680 statistically decreased (*p* < 0.05) when exposed to B:R and B:C mixtures (1.13 × 10^5^ and 2.98 × 10^7^ CFU·mL^−1^, respectively) (Figure 7C). Both strain LMG 2024 and Ea 630 had a decrease in viable cells when exposed to the peptides, but only the R:C mixture caused a statistically significant decrease in LMG 2024 (*p* < 0.05) (Figure 7A,B).

## 3. Discussion

In the last decade, there has been an increasing demand for food availability worldwide, which is going to be further aggravated in the coming years. One major impediment to respond to current and future needs to feed the growing world population regards food losses due to phytopathogens, such as *E. amylovora* [44,45]. To mitigate this problem, and also to respond to the call for a more sustainable agriculture, AMPs have taken the spotlight as good candidates to substitute conventional antibiotics and other chemical compounds with associated health and environmental risks. In a previous study, the bioactivity of five AMPs against *E. amylovora* was determined, alongside the previously studied AMP BP100 [17]. However, only one study has been performed with the equimolar mixture of AMPs [15]. Therefore, the efficacy of three new AMPs mixtures was tested against *E. amylovora*.

MIC and MBC are frequently used to evaluate bacteria susceptibility to antimicrobial compounds, namely of AMPs [24,46,47]. In this study, MIC values showed that the B:R mixture was the most efficient, since it presented inhibitory action at lower concentrations (2.5 μM) for the three strains when compared to B:C and R:C, despite the virulence of each strain. These results were also observed for their MBC values, which allowed us to distinguish that the best AMP mixture was B:R, followed by B:C and then by R:C, as its MBC and MIC values were the same whatever the strain. In contrast, the mixture B:C had an MBC value of 4 μM against Ea 630 which is a highly virulent strain and 3.25 μM against Ea 680, a milder virulent strain, whilst the results were the inverse for R:C for the same strains.

When compared with the MIC and MBC of these strains against the individual AMPs determined previously (Table S1) [17], there was a reduction of the concentration needed to impair growth of bacteria (bacteriostatic effect) and eliminate (bactericidal effect) the bacterial strains tested in an order of 2x or greater, suggesting a possible synergic effect of the AMPs with each other. These results allow us to infer that their combined effect in the membrane of the bacteria could be boosted, leading to lower concentrations required to cause membrane disruption and consequently cell death. The difference of MIC and MBC values between the AMPs mixtures are likely due to their different conformational features, which could influence their mode of action [48]. Interestingly, a recent study tested a mixture of one AMP with a plant defense elicitor peptide (BP16 with flg15) against *E. amylovora*, with a MIC between 12.5 and 25 μM, which is considerably higher than the values herein reported [15].

The IC_50_ values obtained for these combinations against the tested strains, even though without statistical differences, revealed strain-dependent susceptibility, and once more, the inhibitory concentrations were greatly reduced (approximately between 3x and 4x less) when compared with the AMPs individual effects (Table S1) [17]. Still, the same peptide mixture displayed different results between the different strains, which increases the need to expand this study to a larger population of *E. amylovora* to better understand these differences in susceptibility to the same AMPs mixtures.

This decrease of AMPs’ concentration needed to achieve a bactericidal effect could be associated to different affinities with components of the outer cellular membrane of the bacterium, like lipoproteins or liposaccharides [49]. BP100 has great membrane affinity, so its presence in the mixtures might involve its strong binding to the membrane components, allowing the other AMPs to interact more strongly with the inner cell membrane, which has been previously hypothesized for combinations of AMPs with other bactericidal agents [40,49].

Cell viability is one of the most reliable assessments of the efficacy of AMPs, and has been widely applied in diverse studies [17,26,40,50,51,52,53]. However, viability can be attributed to different causes, namely, loss of membrane integrity, reduced metabolism, among other factors, which leads to the need of assessing cell viability by resorting to different techniques. In this study, two assays that have been employed successfully to evaluate AMPs were applied, namely, FC, which allows to assess membrane permeabilization when using a fluorescent dye that, like PI, only emits signal when the cell membrane is compromised, and the second assay was the AB reduction, which evaluates the cell metabolism, based on the reduction of resazurin to resorufin [54].

Regarding FC, it is possible to infer that the AMPs mixtures have the capability to permeate the membrane of the strains tested at the point of contact (t_0_) (Figure 2), with strain Ea 630 presenting the lower percentage value of viability when exposed to B:C. Furthermore, the effect of the AMPs improves greatly after 10 min of exposure with viability decreasing below 50% for every strain, with a steady decrease until two hours of exposure below 25%, which allows to infer that these AMPs possess a quick action in permeating the cell membranes, and that in few hours it could lead to total cell death. This is especially true for the combination of R:C that, after two hours of exposure, there was no cells growth after 24 h when plated in growth medium, as it is possible to observe in CFU plate counting. When comparing these results with the effects of the individual peptides in previous works, it is possible to discern that with lower concentrations (2.5 to 4 μM), these mixtures induce faster and more extensive membrane permeabilization, leading to cell death, while individually similar percentages were observed only when exposed for 2 h at CA-M peptide at 5 and 8 μM (Table S1) [17,40].

The AB assay was useful to further corroborate the FC results, where cells had a great decrease of their metabolic activity after initial exposure to the AMPs mixtures (t_0_) (Figure 4), especially for strains Ea 630 and 680, which decreased their metabolism below 22% when exposed to B:R and B:C mixtures. Metabolism was reduced in the first 30 min of exposure to the AMPs for the three strains, and this decreased remained stable up to 2 h of the assay, confirming the antibacterial efficiency of these AMPs. When cell metabolism is compromised, cell death occurs, and these results further corroborate the hypothesis of these mixtures presenting a synergic action, since they cause faster cell destabilization at lower concentrations, compared to previously reported data on the individual use of BP100, where a concentration of 10 μM was required to achieve similar metabolism reduction [40]. Interestingly, these results agreed with CFU plate counting, which showed a greater decrease of viable cells after 2 h of exposure to the AMPs mixtures for this assay when compared to the CF assay, principally for strain Ea 680 that after exposure to B:R and R:C mixtures, displayed no cell growth. This could be due to some expected variability of viable cells that happens during the necessary procedures to perform each assay.

Viability assays allowed to disclose that the effects of these mixtures are similar in strains with different virulence fitness, and are time dependent, i.e., their bioactivity against this pathogen increases with time, with a high decrease of cell viability observed within a few minutes, and with a clear synergic action. This is consistent with a mode of action for these AMPs that involves significant membrane destabilization, which increases over time as peptide molecules accumulate on the membrane [55]. Moreover, cell death associated to reduced metabolism capacity, associated to irreversible cell membrane damage as assessed by FC, supports the hypothesis that bactericidal action could be reached at lower peptide concentrations when using AMPs mixtures, which is in line with previous findings by Cabrefiga and Montesinos [40].

Once the fast and potent action of the AMPs mixtures against *E. amylovora* was confirmed in vitro, the effects of the mixtures were tested *in planta*, after 60 min of exposure of each AMP mixture to each of the three bacterial strains. It was possible to discern different degrees of infection severity according to the combination of AMPs and the strains tested (Figure 6). The most representative result was obtained with the R:C mixture, which prevented necrosis at the infection site in all the strains (severity scale 0), reinforcing in vitro data that showed this combination to cause cell death within 2 h of exposure. Further, these results were in line with CFU plate counting data, which once again showed that no viable cells were present after 24 h for strains LMG 2024 and Ea 680, in agreement with results from FC (both strains) and AB (latter strain) assays.

Furthermore, the distinct HR severity degrees found after treatment with B:R and B:C mixtures could be due to the different degrees of virulence of each strain, and to the effect of the AMPs on such virulence, since it has been previously proven that peptides can interfere quorum sensing signaling and/or modulate the virulence in Gram-negative bacteria, disrupting the formation of biofilms that are important virulence factors in *E. amylovora* [56,57].

Additionally, HR bioassays allowed to infer on the low or undetectable toxicity of the AMPs tested, since no or low necrosis could be observed after 1 h of exposure to the peptide mixtures. Similar assays have been previously performed to assess the toxicity and/or bactericidal efficacy of AMPs against phytopathogenic bacteria [16,40,46,58]. Synergetic effects in combinations of AMPs with coadjuvants, resulting in low necrosis, had previously been advanced [40], which supports our findings.

Overall, this study demonstrates that the antimicrobial activity of the three tested AMPs is higher when they are combined together in equimolar proportion. This is probably caused by a mutual boost of the mode of action associated to each individual peptide, which mainly concerns destabilization of the bacterial cell membrane. Relevantly, the mixture of short peptide sequences is advantageous over long and/or structurally complex AMPs, since the simpler and shorter a peptide is, the easier, faster, and more cost-effective is its synthesis [46,57,59]. Moreover, using a mixture of different AMPs will probably constrain the emergence of resistant bacteria, as previously advocated [60]. On the other hand, our findings show that the antibacterial efficacy is similar amongst different strains of *E. amylovora*, which suggests that these AMPs mixtures enclose the potential to offer equally good outcomes against other plant pathogenic bacteria.

## 4. Materials and Methods

### 4.1. Peptide Selection and Synthesis

A previous study with six AMPs, on a collection of 36 *E. amylovora* strains collected in Portugal, demonstrated that BP100, RW-BP100 and CA-M peptides (Table 1) were the most efficient in inhibiting *E. amylovora* growth and reducing its viability at concentrations ranging from 5 to 8 μM [17]. As such, an equimolar mixture of these AMPs was performed in a 1:1 (v:v) final ratio as follows: BP100:RW-BP100 (B:R); BP100:CA-M (B:C); RW-BP100:CA-M (R:C).

BP100, RW-BP100 and CA-M peptides (Table 1) were assembled by solid-phase peptide synthesis on an automated Symphony X synthesizer from Gyros Protein Technologies (Tucson, AZ, USA), at the Laboratory of Peptide and Peptide-Nucleic Acid Synthesis of the Faculty of Sciences of the University of Porto (POP-UP), and following an orthogonal Fmoc/tBu scheme [43] using a Fmoc-Rink-amide MBHA resin (100–200 mesh, 0.36 mmol.g-1-NovaBiochem, Merck KGaA, Darmstadt, Germany). All peptides presented a purity degree >98% that was quantitated by analytical reverse-phase high performance liquid chromatography (RP-HPLC) using a Hitachi-Merck LaChrom Elite system equipped with a quaternary pump, a thermostated automated sampler, and a diode-array detector (DAD). Analyses were performed with a reverse-phase C18 column (150 × 4.6 mm ID and 5 μM pore size, Merck) at a 1 mL/min flow rate using a 1–100% of solvent B (ACN) in solvent A, for 30 min, with detection at 220 nm. An LCQ-DecaXP LC-MS system from ThermoFinnigan, equipped with both a DAD detector and an electrospray ionization-ion trap mass spectrometer (ESI/IT MS) was used to confirm peptide identity. RP-HPLC chromatograms and ESI-IT MS spectra of synthesized peptides are provided as Supplementary Information (Figures S2–S7).

### 4.2. Bacterial Strains and Conditions

Two *E. amylovora* strains (Ea 630 and Ea 680), isolated from pear and apple orchards from the center of Portugal in 2015, were evaluated (Table 3). These strains were selected based on their genotypic and phenotypic characteristics; Ea 630 was the most and Ea 680 the least virulent from a collection of 36 *E. amylovora* Portuguese strains [6], showing different susceptibility to BP100, RW-BP100 and CA-M peptides [17]. Type strain LMG 2024 was used as a reference. Bacterial strains were preserved at −80 °C in 30% glycerol and 70% King’s B (KB) medium. Strains were cultured in KB medium at 28 °C.

### 4.3. Bacterial Susceptibility to AMPs Mixtures

To assess the susceptibility of three strains of *E. amylovora* (LMG 2024, Ea 630, and Ea 680) against the three AMPs mixtures it was performed the minimum inhibitory concentration (MIC), minimum bactericidal concentration (MBC) and half maximal inhibitory concentration (IC_50_), using the following concentrations for the final equimolar (1:1) AMPs mixtures: 0.25, 1, 1.75, 2.5, 3.25, 4, 5 and 6 μM.

For MIC assays, bacterial strains were cultured in 2x Mueller Hinton (MH) medium at 25 °C and 180 rpm during 16 h and then adjusted to a final absorbance of 0.08 (approximately 10^8^–10^9^ CFU·mL^−1^) at 600 nm (OD600). The bacterial suspensions were added to a 96-well microtiter plate to a final volume of 150 μL per well (75 μL of bacterial culture and 75 μL of AMP mixture, in order to obtain a final ratio of 1:1). The microtiter plate was then placed in a multiplate spectrophotometry reader (Multiskan™ GO, Thermo Fisher Scientific, Massachusetts, USA) at 25 °C. After 24 h, the absorbance was measured at 600 nm, and results were obtained when growth inhibition was detected. Following, 10 μL of bacterial suspension of each well were grown in triplicate in a petri dish containing KB medium, at 25 °C for 24 h, and MBC was obtained when there was no visible colony growth. In order to further evaluate the susceptibility of these strains against the three AMPs mixtures, especially in those where the MIC and MBC results were the same, the IC_50_ was determined through nonlinear fitting, resorting to GraphPad Prism 9 software (GraphPad Software, San Diego, CA, USA). For every susceptibility assay, sterile deionized water (H2Od), and chlortetracycline (50 μM) were used as positive and negative control, respectively. Each assay was performed at least three times.

### 4.4. Bacterial Viability Assays

In order to evaluate the *E. amylovora* strains’ viability after exposure to the three AMPs mixtures tested, a two-way approach was employed, namely the evaluation of the membrane permeability through flow cytometry (FC), and the quantification of cell metabolism using the alamarBlue™ (AB) reduction method. The concentrations used for both analyses where those obtained in the MBC assays.

#### 4.4.1. Membrane Permeability Analysis

FC analysis was employed to evaluate *E. amylovora* strains (Ea 630 and Ea 680) and type strain LMG 2024 membrane integrity after exposure to the AMPs mixtures, using propidium iodide (PI) as fluorochrome. Briefly, the bacterial strains were grown in KB medium for 24 h, with constant agitation at 180 rpm at 25 °C; a bacterial pellet was then obtained through centrifugation and resuspended in sterile phosphate buffer solution (PBS, pH 7.2, 10 mM). OD600 was adjusted for each strain at 0.08, and 50 μL of the inoculum was exposed to each AMP mixture and stained with PI in a final concentration of 1 mg.mL^™1^. Fluorescence intensities were recorded at t_0_, t_10_, t_30_, t_60_, and t_120_ min. The analysis was performed on a BD Accuri™ C6 Plus flow cytometer (BD Bioscience, NJ, USA). Data were collected for each assay at 20,000 events and assessed by gating using flow cytometry software C6 Plus Analysis (BD Bioscience, East Rutherford, NJ, USA). Membrane permeability was directly proportional to PI fluorescence intensity. H2Od and isopropyl alcohol 23% were used as negative and positive control, respectively. Each experiment was repeated at least three times.

#### 4.4.2. alamarBlue™ Cell Viability Assessment

To further evaluate *E. amylovora* strains and type strain LMG 2024 viability after exposure to the AMPs mixtures, quantification of the metabolism of the cells was determined resorting to alamarBlue™ Cell Viability Reagent (Thermo Fisher Scientific, Waltham, MA, USA), as described by the manufacturer. Briefly, bacteria cultures of the *E. amylovora* strains and type strain were grown as described in Section 4.4.1 and, after centrifugation, the pellet was resuspended in PBS (pH 7.2, 10 mM) and OD600 was adjusted to 0.08. These bacterial inoculums were later exposed to the different concentrations of each AMP mixture, and incubated for two h at 25 °C. Quantification of cell metabolism was performed at t_0_, t_10_, t_30_, t_60_, and t_120_ min of exposure to the AMPs mixtures. For that, 10 μL of the suspension was placed in triplicate in a 96 well titration plate, and 90 µL of alamarBlue™ Cell Viability Reagent was added, and the plate was incubated in the dark at 37 °C for 4 h. Samples’ absorbance was measured in a Multiskan™ GO, at 570 and 600 nm. The number of viable cells correlates with the amount of dye reduction and is expressed as the percentage of AB reduction (%AB reduction). The percentage of AB reduction was calculated based on the following equation.

$$\%\mathrm{AB}\;\mathrm{reduction}=\frac{\left(\varepsilon_{\mathrm{ox}\;}\lambda_{2}\right)\left({A\lambda }_{1}\right)-\left(\varepsilon_{\mathrm{ox}\;}\lambda_{1}\right)\left({A\lambda }_{2}\right)}{\left(\varepsilon_{\mathrm{red}}\lambda_{1}\right)\left({A\prime \lambda }_{2}\right)-\left(\varepsilon_{\mathrm{red}\;}\lambda_{2}\right)\left({A\prime \lambda }_{1}\right)}\;\times \;100$$

$\varepsilon_{\mathrm{ox}\;}\lambda_{1}$—molar extinction coefficient of oxidized AB at 570 nm;

$\varepsilon_{\mathrm{ox}\;}\lambda_{2}$—molar extinction coefficient of oxidized AB at 600 nm;

$\varepsilon_{\mathrm{red}}\lambda_{1}$—molar extinction coefficient of reduced AB at 570 nm;

$\varepsilon_{\mathrm{red}}\lambda_{2}$—molar extinction coefficient of reduced AB at 600 nm;

${A\lambda }_{1}$—absorbance of test well at 570 nm;

${A\lambda }_{2}$—absorbance of test well at 600 nm;

${A\prime \lambda }_{1}$—absorbance of negative control at 570 nm;

${A\prime \lambda }_{2}$—absorbance of negative control at 600 nm.

H_2_Od and isopropyl alcohol 23% were used as negative and positive control, respectively. Each experiment was repeated at least three times.

### 4.5. Hypersensitive Response in Tobacco Plants

In order to evaluate the effects of the infection *in planta* by the three *E. amylovora* strains (Ea 630, Ea 680, and type strain LMG 2024), after exposure to AMPs mixtures, hypersensitive response (HR) was performed in *Nicotiana tabacum*. After analyzing the results of the previous viability assays, it was determined that one h of exposure to the peptides was sufficient to test in plants. The bacterial strains were grown in KB medium for 16 h, at constant agitation of 180 rpm at 25 °C in an incubator, and then, after centrifugation, cell pellets were resuspended in PBS (pH 7.2, 10 mM), and their OD600 was adjusted to 0.08. Next, each strain was exposed to the concentrations determined previously in the MBC, for each peptide, for 1 h. After that, 1 mL of inoculum was inserted in the abaxial side of 10-week-old N. tabacum cv. ‘Virginia Gold’ leaves, with a needless sterile syringe. Results were recorded and photographed after 24 and 48 h after inoculation, and were expressed using an ordinal categorical scale of severity of infection (0 to 3) as described by Cabrefiga and Montesinos [40], namely, 0, no symptoms; 1, leaf necrosis localized around the wound; 2, necrosis progression far from the wound; 3, necrosis extended to most part of the whole leaf. PBS was used as negative control. All assays were made at least three times in triplicates, i.e., three N. tabacum plants were used, three infection points per leaf. Individual AMPs were also tested.

### 4.6. Assessment of Colony Forming Units (CFU)

The number of viable cells after treatments with each AMP mixture, for both the viability, and HR assays, were estimated by determining the number of colony forming units (CFU). Following the time-controlled exposure of the bacteria to the different AMPs mixtures and concentrations defined for each assay, tenfold dilutions series were prepared and 10 μL of each dilution and the original sample was culture in triplicate in KB medium, at 25 °C. After 24 h incubation the number of CFUs were determined. These assays were repeated at least three times.

### 4.7. Statistical Analysis

Comparisons between the treatments for IC_50_ and CFU were analyzed with one-way ANOVA, whilst FC and AB were analyzed with two-way ANOVA using GraphPad Prism 9 for Windows (GraphPad Software, CA, USA). Results were considered statistically different when *p* < 0.05.

## 5. Conclusions

The findings of this work show the synergetic effects arising from mixing different AMPs at equimolar amounts, making the mixtures thus obtained suitable candidates for eco- and biofriendly control measures of *E. amylovora* alongside the preventive measures currently applied, hence contributing towards a greener agriculture.

Furthermore, our findings reveal that, even for more virulent strains, the AMP mixtures tested significantly reduced bacterial cell viability with half or less the concentration needed when using the individual peptides alone. This was accompanied with reduced HR effects *in planta*, especially in the case of the RW-BP100:CA-M combination.

In the future, additional studies should be conducted with these AMPs, covering other different combinations (e.g., mixtures with more than two peptides and mixtures of AMPs with other nonpeptidic antimicrobial agents), and also covalent conjugation instead of noncovalent mixtures. Investigation of the peptide effects, alone or combined, on hormonal response and gene expression in the preferred hosts of *E. amylovora*, should also be pursued.

## Figures and Tables

**Table 1:** Sequence and properties of peptides used in this work.

Peptide	Sequence	Net Charge ^a^	MW (Da) ^b^
**BP100**	KKLFKKILKYL-NH_2_	+6	1419.9
**RW-BP100**	RRLFRRILRWL-NH_2_	+6	1583.0
**CA-M**	KWKLFKKIGAVLKVL-NH_2_	+6	1769.2

^a^ Estimated net charge at pH 7; ^b^ MW: molecular weight. Source: Pepdraw.com.

**Table 2:** AMPs mixtures values of minimal inhibitory concentration (MIC), and minimal bactericidal concentration (MBC) obtained for three *Erwinia amylovora* strains.

AMP	Strain	MIC (μM)	MBC (μM)
**BP100:RW-BP100**	LMG 2024	2.5	2.5
	Ea 630	2.5	2.5
	Ea 680	2.5	2.5
**BP100:CA-M**	LMG 2024	2.5	3.25
	Ea 630	4	4
	Ea 680	2.5	3.25
**RW-BP100:CA-M**	LMG 2024	4	4
	Ea 630	3.25	3.25
	Ea 680	2.5	4

**Table 3:** *Erwinia amylovora* strains used in this work.

Strain	Host		Isolated From	Geographic Origin	Year
	Species	Cultivar			
**LMG 2024**	Pear	N/D	N/D	UK	1959
**Ea 630**	Apple	‘Gala’	Branch	Cadaval, Portugal	2015
**Ea 680**	Pear	‘Rocha’	Branch	Cadaval, Portugal	2015

## Supplementary Materials

The following are available online at https://www.mdpi.com/article/10.3390/plants10122637/s1, Table S1: Summary results of effects of individual AMPs used in the study of Mendes et al. [17]. Detailed results can be found in Mendes et al. [17]. Figure S1: Hypersensitive response in tobacco leaves after 24 hours of inoculation of *Erwinia amylovora* type strain exposed to three individual AMPs, namely, BP100, RW-BP100, and CA-M; +: positive control; −: negative control (PBS); HPI: hours post infection. Figure S2: Full ESI-IT MS (positive mode) obtained for peptide BP100 (MW = 1419.9 Da). Figure S3: RP-HPLC chromatogram for peptide BP100, after purification; gradient elution from 1 to 100% ACN in 0.05% aqueous TFA at 1 mL/min flow rate, for 30 min, on a C-18 column (150 × 4.6 mm ID and 5 μM pore size); detection at 220 nm, Figure S4: Full ESI-IT MS (positive mode) obtained for peptide RW-BP100 (MW = 1583.0 Da), Figure S5: RP-HPLC chromatogram for peptide RW-BP100, after purification; gradient elution from 1 to 100% ACN in 0.05% aqueous TFA at 1 mL/min flow rate, for 30 min, on a C-18 column (150 × 4.6 mm ID and 5 μM pore size); detection at 220 nm, Figure S6: Full ESI-IT MS (positive mode) obtained for peptide CA-M (MW = 1769.2 Da), Figure S7: RP-HPLC chromatogram for peptide CA-M after purification; gradient elution from 1 to 100% ACN in 0.05% aqueous TFA at 1 mL/min flow rate, for 30 min, on a C-18 column (150 × 4.6 mm ID and 5 μM pore size); detection at 220 nm.
