# Supplementary material for: Evaluation of Three Antimicrobial Peptides Mixtures to Control the Phytopathogen Responsible for Fire Blight Disease
Source: Plants (Basel). 2021 Nov 30;10(12):2637. doi: 10.3390/plants10122637 (PMC8705937; doi:10.3390/plants10122637)
Supplement: Supplementary file 1 [file plants-10-02637-s001.zip › SF1.pdf]

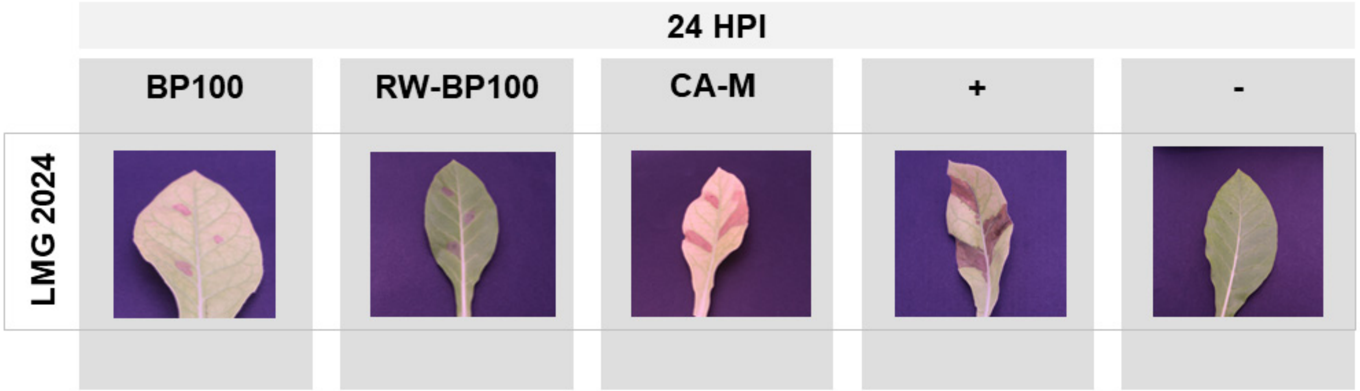

Figure S1. Hypersensitive response in tobacco leaves after 24 hours of inoculation of *Erwinia amylovora* type strain exposed to three individual AMPs, namely, BP100, RW-BP100, and CA-M; +: positive control; -: negative control (PBS); HPI: hours post infection.
