# Supplementary material for: Evaluation of Three Antimicrobial Peptides Mixtures to Control the Phytopathogen Responsible for Fire Blight Disease
Source: Plants (Basel). 2021 Nov 30;10(12):2637. doi: 10.3390/plants10122637 (PMC8705937; doi:10.3390/plants10122637)
Supplement: Supplementary file 1 [file plants-10-02637-s001.zip › SF3.pdf]

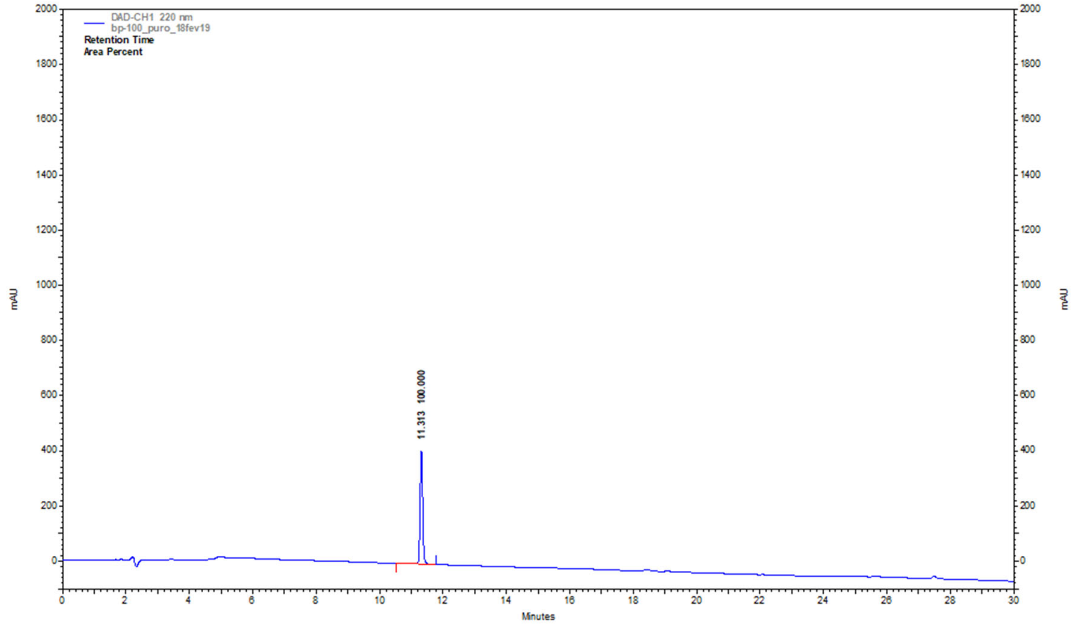

Figure S3. RP-HPLC chromatogram for peptide BP100, after purification; gradient elution from 1 to 100% ACN in 0.05% aqueous TFA at 1 mL/min flow rate, for 30 min, on a C-18 column (150 × 4.6 mm ID and 5 µm pore size); detection at 220 nm.
