# Supplementary material for: Evaluation of Three Antimicrobial Peptides Mixtures to Control the Phytopathogen Responsible for Fire Blight Disease
Source: Plants (Basel). 2021 Nov 30;10(12):2637. doi: 10.3390/plants10122637 (PMC8705937; doi:10.3390/plants10122637)
Supplement: Supplementary file 1 [file plants-10-02637-s001.zip › SF4.pdf]

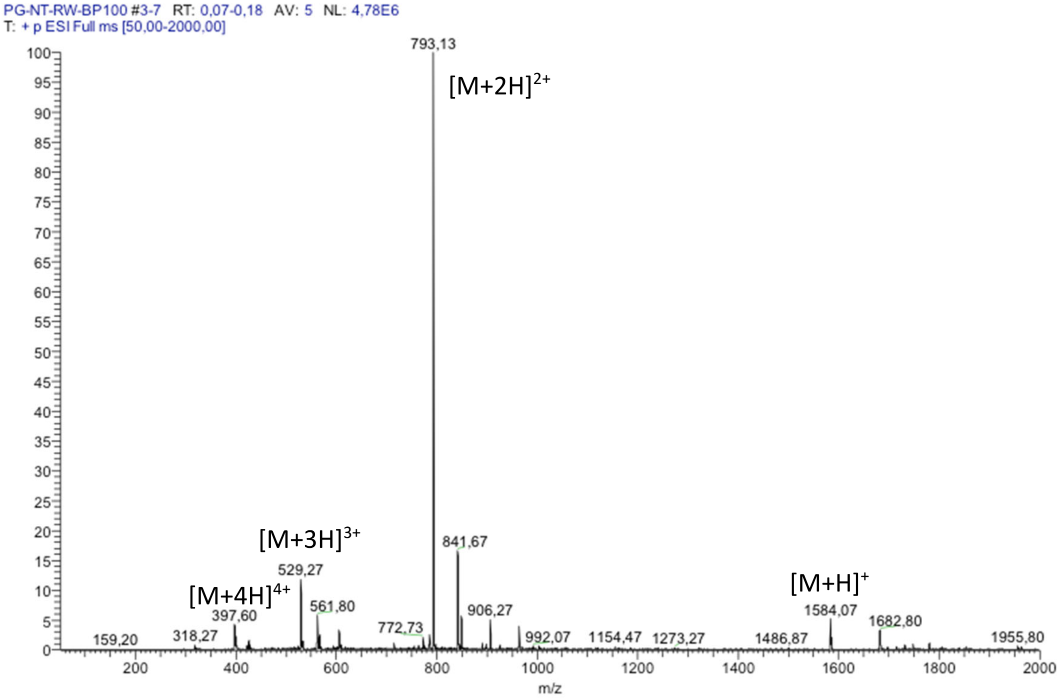

Figure S4. Full ESI-IT MS (positive mode) obtained for peptide RW-BP100 (MW=1583.0 Da).
