# Supplementary material for: Evaluation of Three Antimicrobial Peptides Mixtures to Control the Phytopathogen Responsible for Fire Blight Disease
Source: Plants (Basel). 2021 Nov 30;10(12):2637. doi: 10.3390/plants10122637 (PMC8705937; doi:10.3390/plants10122637)
Supplement: Supplementary file 1 [file plants-10-02637-s001.zip › ST1.pdf]

Table S1. Summary results of effects of individual AMPs used in the study of Mendes *et al*. [17]. Detailed results can be found in Mendes *et al*. [17].

AMP	Strain	MIC (µM)	MBC (µM)	IC_{50} (µM)	FC (5 / 8 µM) (%) [a]	UFC (5 / 8 µM) (Log_{10} ($UFC.mL^{-1}$)) [a]
**BP100**	LMG 2024	5	8	3.045 ± 0.53	91.9 / 73.7	8.111 / 7.599
	Ea 630	8	8	4.531 ± 1.09		
	Ea 680	8	8	3.151 ± 0.460		
**RW-BP100**	LMG 2024	5	5	3.506 ± 0.07	92.2 / 89.1	0 / 0
	Ea 630	5	5	3.478 ± 0.08		
	Ea 680	5	5	3.522 ± 0.06		
**CA-M**	LMG 2024	8	8	4.851 ± 0.66	12.1 / 2.3	0 / 0
	Ea 630	8	8	4.747 ± 0.80		
	Ea 680	5	5	3.496 ± 0.09		

[a] Results presented here pertain to 2 hours of exposure to AMPs
